# Clinical Nursing Service Value: A Hybrid Concept Analysis

**DOI:** 10.1155/jonm/7028770

**Published:** 2026-10-23

**Authors:** Pei Chen, Xueqing Zhang, Yilin Chen, Ben Yang, Mingqiu Gu, Guihua Xu, Yamei Bai, Yulei Song

**Affiliations:** ^1^ School of Nursing Nanjing University of Chinese Medicine Nanjing China njucm.edu.cn; ^2^ Jiangsu Provincial Key Laboratory of Critical Care Medicine Department of Critical Care Medicine, Zhongda Hospital Southeast University Nanjing China seu.edu.bd

**Keywords:** clinical nursing service value, concept analysis, nursing, value-based health care

## Abstract

**Aim:**

To clarify the attributes, antecedents, and consequences of clinical nursing service value.

**Background:**

Nursing service constitutes a core component of the healthcare system, significantly influencing patient outcomes and system performance. However, the value of nursing service remains inadequately identified and measured. With the shift toward value‐based healthcare, it is urgent to clarify the connotation of clinical nursing service value. Existing research lacks an integrative framework to comprehensively capture the unique contributions of nursing services.

**Methods:**

The hybrid concept analysis model was employed. In the theoretical phase, a systematic search was conducted across multiple databases, including PubMed, Web of Science, MEDLINE Complete, CINAHL Complete, Embase, Emerald Insight, China National Knowledge Infrastructure, Wanfang Data Knowledge Service Platform, Chinese Science and Technology Journals Database, and China Biology Medicine disc. The literature search was updated through September 14, 2026, and 43 eligible articles were included and analyzed. In the fieldwork phase, 27 patients, 10 nurses, and 3 nurse managers were selected for semistructured interviews, and the interview data were analyzed by conventional content analysis. In the analytical phase, findings from both the theoretical and fieldwork stages were integrated to derive the definitive conceptual definition.

**Results:**

Clinical nursing service value is a composite value system comprising five interconnected dimensions: technical, economic, humanistic, informational, and social. Its proposed antecedents span system and organization, the nursing team, patients and families, and technology and information, with consequences identified for patients, nurses and the nursing profession, hospitals, and the healthcare system.

**Conclusion:**

This study constructs a comprehensive conceptual model of clinical nursing service value. It provides a theoretical framework for identifying, measuring, and enhancing nursing value and offers a crucial foundation for the nursing profession to secure policy and resource support.

**Implications for Nursing Management:**

This conceptual framework may inform nursing performance evaluation, workforce planning, and resource allocation. Empirical validation is required before its use in appraisal, reimbursement, or compensation decisions.

## 1. Introduction

Nursing service constitutes an indispensable and core component of the healthcare system. Globally, nurses represent the largest proportion of health professionals [1]. Their work is closely linked to patient health outcomes, societal health levels, and the performance of healthcare systems [2, 3]. The International Nurses Day 2024 report states that nursing is not merely a cost within healthcare services, but a crucial investment capable of generating significant value [1, 4].

However, a persistent paradox exists: While the importance of clinical nursing is universally acknowledged, its comprehensive value remains inadequately recognized and measured within management, policy, and societal spheres. The economic value of nursing is often hidden in bundled payments [5], while nonquantifiable aspects like professional judgment and compassionate care are frequently undervalued [6]. This ambiguity has significant repercussions. In practice, the contributions of nursing staff often lack sufficient environmental support or performance feedback, which can undermine work motivation and exacerbate professional burnout [7]. At the management and policy levels, the failure of full value recognition marginalizes nursing in resource and policy decisions, ultimately limiting its potential to enhance healthcare efficiency [8].

With the rise of the “Value‐Based Healthcare” concept, the goal of healthcare is shifting toward creating comprehensive health value centered on the patient, emphasizing optimized health outcomes while controlling costs [9]. This paradigm shift necessitates a systematic and structured conceptual clarification of clinical nursing service value. The pursuit of defining clinical nursing service value has evolved alongside healthcare delivery models. Initial research introduced the concept of “value‐based healthcare” into nursing, defining nursing value as patient outcomes achieved per unit of nursing cost [10]. This perspective highlighted cost‐effectiveness but proved reductive. Subsequent research recognized that nursing value encompasses not only economic aspects but also substantial intangible assets that are difficult to monetize [11].

As research advanced, conceptual studies on nursing service value have shown a multidimensional expansion, extending its connotations to technical, preventive, and social dimensions [12]. Further analysis from a stakeholder perspective revealed divergent value perceptions: Payers focus on cost‐effectiveness, nurses on perceived professional worth, and patients on emotional support within the therapeutic relationship [13]. Human capital theory, production theory, and value theory have also been introduced to the exploration of nursing value. Within these theoretical frameworks, nursing is recognized as a form of appreciating human capital, the value of which can be considered and measured from a return on investment (ROI) perspective [14].

Although previous studies have conceptualized nursing value, most have relied on literature‐based approaches or focused on selected dimensions. Limited attention has been paid to integrating perspectives from patients, nurses, and nurse managers in clinical practice. Existing studies have tended to emphasize measurable economic outcomes or isolated professional contributions, whereas the cognitive, humanistic, informational, and social contributions embedded in routine clinical nursing work remain insufficiently conceptualized. This limitation is particularly important in China, where many nursing activities are incorporated into bundled service charges and are not independently represented in service pricing, performance evaluation, or workforce planning. As a result, the professional contribution of nursing is often treated as a cost rather than as a value‐generating component of healthcare delivery.

A rigorous concept analysis is therefore needed to clarify what constitutes clinical nursing service value, how it is generated, and what outcomes it produces. The hybrid concept analysis model was selected because clinical nursing service value is both theoretically underdeveloped and highly context‐dependent. Purely literature‐based approaches to concept analysis, such as traditional or evolutionary concept analysis, are useful for clarifying how a concept has been described in existing scholarship, but they may insufficiently capture how the concept is experienced, enacted, and interpreted in clinical practice. The hybrid model, by combining a theoretical phase, a fieldwork phase, and a final analytic phase, enables the integration of published evidence with the perspectives of patients, nurses, and nurse managers [15]. This approach was therefore particularly suitable for developing a concept that is both theoretically grounded and practically meaningful for nursing management and policy.

Clarifying clinical nursing service value has important implications for nursing policy, management, and workforce planning. At the practice and management levels, it provides a basis for making nursing contributions identifiable, measurable, and rewardable, thereby supporting multidimensional performance evaluation and more appropriate nursing resource allocation. At the policy level, it may inform value‐based reforms in nursing service pricing, medical insurance payment, and workforce planning. In this sense, the proposed model supports the nursing profession by transforming nursing work from an invisible and undervalued contribution into a professional value system that can be articulated, evaluated, and strengthened.

Therefore, this study aimed to extend prior conceptualizations of nursing value through a hybrid, multistakeholder concept analysis and to clarify its attributes, antecedents, consequences, and definition.

## 2. Method

### 2.1. Study Design

The hybrid concept analysis method was utilized to define the concept of clinical nursing service value. The method comprises three phases: theoretical phase, fieldwork phase, and analytic phase [16]. We followed the Preferred Reporting Items for Systematic Reviews and Meta‐Analysis 2020 guidelines [17] during the theoretical phase and adhered to the Consolidated Criteria for Reporting Qualitative Research (COREQ) checklist [18] during the fieldwork phase (see Supporting File S1).

### 2.2. Theoretical Phase

In this phase, a systematic literature review was conducted to identify how clinical nursing service value has been described, defined, and operationalized in previous studies. Searches were performed in both international and Chinese databases, including PubMed, Web of Science, MEDLINE Complete, CINAHL Complete, Embase, Emerald Insight, China National Knowledge Infrastructure (CNKI), Wanfang Data Knowledge Service Platform (Wanfang Data), Chinese Science and Technology Journals Database (VIP), and China Biology Medicine disc (CBM). The search covered articles published before December 30, 2024.

Search terms included combinations of nursing‐related terms (“nursing,” “nurse,” “nurs∗,” “care,” and “nursing service”) and value‐related terms (“service value,” “value,” and “professional value”). The search strategy combined database‐specific subject headings, where available, with free‐text terms, and was supplemented by a title‐based search to balance sensitivity, precision, and reproducibility, consistent with established recommendations for systematic search strategy development and reporting [19]. The supplementary title‐based search (nurs∗[Title] AND value[Title]) was used to capture articles using alternative expressions such as “nursing value” or “value of nursing” as broad retrieval is recommended in concept analysis to ensure conceptual saturation [15]. The title restriction was applied to maintain precision and reduce the retrieval of records in which “value” was mentioned only peripherally. The full database‐specific search strategies are provided in Supporting File S2. In the Chinese academic context, the term “professional value” may be used not only to describe nurses’ personal professional values, but also to discuss the contributions, role, recognition, and social significance of nursing services or the nursing profession. Therefore, “professional value” was retained as an additional free‐text term to improve retrieval sensitivity. During screening, studies retrieved by this term that primarily addressed nurses′ personal professional values, professional beliefs, ethical attitudes, or professional value scales were excluded to maintain conceptual focus.

The inclusion criteria were as follows: (1) published journal articles; (2) publications in English or Chinese; (3) full‐text availability; and (4) relevance to clinical nursing service value, including extractable information on at least one attribute, antecedent, or consequence. The exclusion criteria were as follows: (1) Studies set in nonclinical nursing contexts; (2) studies focusing primarily on nurses’ professional values rather than the value generated by clinical nursing services; and (3) letters, editorials, news reports, comments, expert lectures, and conference abstracts.

Because the theoretical phase aimed to clarify the conceptual use, meaning, and dimensional structure of clinical nursing service value rather than to synthesize intervention effects or estimate outcome evidence, methodological quality was not used as an exclusion criterion. Instead, two reviewers independently conducted a conceptual relevance appraisal by assessing whether each article explicitly discussed clinical nursing service value and provided extractable information on its attributes, antecedents, or consequences. Studies with weaker methodological designs were retained when they contributed conceptually relevant meanings, as excluding them could have narrowed the conceptual scope of the analysis.

The initial search identified 12,849 records. After duplicate removal, 6591 records remained for title and abstract screening. A total of 274 full‐text articles were assessed for eligibility, and 38 studies were finally included in the qualitative synthesis (21 in English and 17 in Chinese) (Figure 1). Prior to the final submission, we updated the search strategy by rerunning the same search protocol across all databases. The final search was conducted on September 14, 2026. Five additional articles met the inclusion criteria and were incorporated into the final analysis, leading to a total of 43 articles included in the theoretical phase. The five newly included studies were consistent with the existing categories and did not alter the five‐dimensional model, definition, antecedents, or consequences.

**FIGURE 1:**
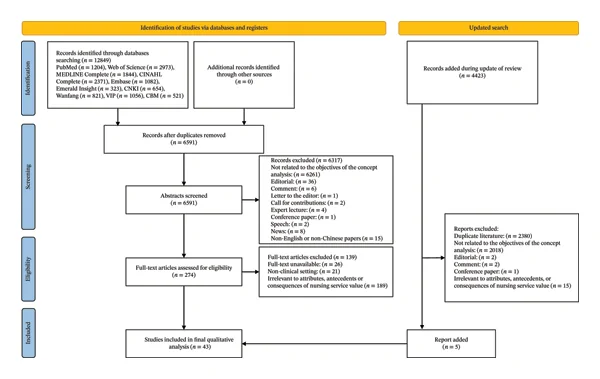
PRISMA 2020 flow diagram for the initial and updated searches in the theoretical phase.

Data were extracted using a structured review sheet, including author, publication year, country, study design, methodological characteristics, and descriptions of attributes, antecedents, and consequences (Supporting File S3). One reviewer [Pei Chen] performed the initial extraction and coding, which was independently checked by another reviewer [Yilin Chen]. Disagreements were discussed with a third reviewer [Xueqing Zhang] until consensus was reached.

### 2.3. Fieldwork Phase

In this phase, a qualitative study was conducted to explore participants’ experiences and perceptions of clinical nursing service value. Purposive sampling was used to recruit participants with direct experience of receiving, providing, or managing clinical nursing services, and maximum variation sampling was applied to capture diverse perspectives across demographic, professional, and clinical characteristics.

Participants were recruited from two tertiary Grade A hospitals in East China. Within this setting, we sought variation across departments, clinical roles, professional titles, and years of experience. Nurses and nurse managers were recruited from medical and surgical units, including respiratory and critical care medicine, cardiology, endocrinology and metabolism, general surgery, gastrointestinal and oncologic surgery, and orthopedics. Patients were recruited with attention to age, gender, education level, disease category, treatment type, and length of hospital stay.

To reduce sampling bias, the research team periodically reviewed participant characteristics during recruitment and conducted targeted recruitment when certain subgroups were underrepresented. Ultimately, 27 patients, 10 clinical nurses, and 3 nurse managers participated in the interviews on the attributes, antecedents, and consequences of clinical nursing service value (Tables 1 and 2).

**TABLE 1:** Demographic statistics of patient participants (*n* = 27).

Characteristic	Category	*N* (%)
Patients (*N* = 27)		

Gender	Female	14 (51.85)
	Male	13 (48.15)

Age (years)	20–39	4 (14.81)
	40–59	5 (18.52)
	60–79	15 (55.56)
	80–99	3 (11.11)

Education level	Primary education and below	5 (18.52)
	Secondary education	13 (48.15)
	Undergraduate education	8 (29.63)
	Graduate education	1 (3.70)

Marital status	Married	25 (92.59)
	Unmarried	2 (7.41)

Treatment	Surgical	6 (22.22)
	Nonsurgical	21 (77.78)

Disease diagnosis	Circulatory system	5 (18.52)
	Respiratory system	6 (22.22)
	Endocrine system	8 (29.63)
	Digestive system	7 (25.93)
	Urinary system	1 (3.70)

Length of service (days)	1–10	21 (77.78)
	11–20	5 (18.52)
	21–30	1 (3.70)

**TABLE 2:** Demographic statistics of nurse and nurse manager participants (*n* = 13).

Characteristic	Category	*N* (%)
Gender	Female	12 (92.31)
	Male	1 (7.69)

Age (years)	20–39	6 (46.15)
	40–59	7 (53.85)

Education level	Undergraduate education	11 (84.62)
	Graduate education	2 (15.38)

Marital status	Married	9 (69.23)
	Unmarried	4 (30.77)

Role	Clinical nurse	10 (76.92)
	Nurse manager	3 (23.08)

Professional title	Senior nurse	4 (30.77)
	Supervisor nurse	4 (30.77)
	Associate chief nurse	3 (23.08)
	Chief nurse	2 (15.38)

Department	Respiratory and Critical Care Medicine	2 (15.38)
	Cardiology	3 (23.08)
	Endocrinology and Metabolism	3 (23.08)
	General Surgery	1 (7.69)
	Gastrointestinal and Oncologic Surgery	2 (15.38)
	Orthopedics	2 (15.38)

Clinical experience (years)	1–10	4 (30.77)
	11–20	6 (46.15)
	> 20	3 (23.08)

*Note:* In reporting findings from the fieldwork phase, participants are referred to using the format [Role Abbreviation][Sequence Number], where *P* represents patient, *N* represents nurse, and *M* represents nurse manager.

Data were collected through semistructured, face‐to‐face interviews. Prior to the formal interviews, we conducted a preinterview with three participants and subsequently defined the core questions as follows: “In your view, what is nursing service value?” “Can you describe a specific, memorable instance where you observed the nursing service value?” “What factors do you believe facilitate or restrict the realization of nursing service value?” and “What have been the resulting impacts of achieving nursing service value?” Each interview was held in the sole presence of the researcher and the participant, lasting for approximately 30 to 60 min. All interviews were conducted in Chinese, audio‐recorded, and transcribed verbatim. Representative quotations selected for the English‐language manuscript were independently translated into English by two bilingual researchers and verified by consensus.

The interviews were conducted by two researchers (Pei Chen and Yilin Chen). Both researchers were female Master of Science in Nursing candidates who had completed formal coursework in qualitative research methodology. This training provided them with foundational skills in interviewing techniques and data collection.

Data analysis was conducted concurrently with data collection using conventional content analysis according to Graneheim and Lundman’s approach [20]. NVivo 12 was used to manage transcripts, codes, and categories. Two researchers [Pei Chen and Yilin Chen] independently read the transcripts line by line, identified meaning units, condensed them, and generated initial codes inductively. Similar codes were compared and grouped into subcategories, which were further synthesized into categories related to the attributes, antecedents, and consequences of clinical nursing service value.

The two researchers cross‐checked all coding results. Discrepancies were resolved by returning to the original transcripts and, when necessary, by discussion with a third researcher [Xueqing Zhang] until consensus was reached. Coding decisions, category revisions, and disagreement‐resolution processes were recorded in a research log to form an audit trail. Two additional researchers [Xueqing Zhang and Yulei Song] reviewed selected transcripts and category structures to enhance confirmability. Data collection and preliminary analysis proceeded iteratively. Saturation was assessed across the complete sample rather than separately within participant subgroups. After every 3–5 interviews, the research team reviewed whether new codes or categories had emerged. Data collection was considered sufficient when three consecutive interviews generated no new codes and the existing categories adequately explained the data [21]. Subgroup‐specific saturation was not claimed. A coding example showing the progression from a meaning unit to code, subcategory, and category is provided in Supporting File S4.

### 2.4. Analytic Phase

In the analytic phase, findings from the theoretical and fieldwork phases were compared and integrated. Similarities and differences between literature‐derived and fieldwork‐derived categories were examined, and overlapping or conceptually related categories were synthesized to generate the final attributes, antecedents, and consequences of clinical nursing service value.

### 2.5. Ethical Considerations

The Ethics Committee of Nanjing University of Chinese Medicine approved this study (code: 2025NZY‐22‐01). Ethical approval was obtained on July 16, 2025, before participant recruitment. Interviews were conducted between July 20, 2025, and August 20, 2025. This study was conducted in accordance with the principles of the Declaration of Helsinki.

Prior to the interviews, researchers detailed the study’s purpose and procedures, informed participants about audio recording, and emphasized their right to refuse or withdraw without consequences. Written informed consent was obtained thereafter. Interview recordings were transcribed by research team members, with all identifying information replaced by codes. All personal identifiers were anonymized in the transcripts, and data were stored securely to ensure confidentiality.

### 2.6. Rigor

The study applied the criteria proposed by Guba and Lincoln to ensure the credibility, confirmability, transferability, and dependability of the data [22]. Credibility was established through prolonged engagement with the research and data triangulation. Confirmability was achieved by inviting experts to review the raw data, codes, and categories. Dependability was ensured by providing a comprehensive description of the research methods and procedures.

## 3. Results

### 3.1. Findings of Theoretical Phase

#### 3.1.1. Attributes of Clinical Nursing Service Value

The systematic literature review revealed five distinct categories describing the concept of clinical nursing service value (Table 3).

**TABLE 3:** Concept analysis of clinical nursing service value in the theoretical phase.

The phase of the study	Categories	Subcategories
Attributes	Technical value	Accurately assess and identify nursing issues
		Develop and implement individualized nursing plans
		Perform safe and effective technical procedures
		Conduct surveillance and provide early warning
		Provide comfort care
	Economic value	Cost containment
		Efficiency improvement
		Avoidance of preventable resource consumption
		Return on health investment
	Humanistic value	Treat patients with respect
		Provide psychological support to patients
		Create a positive care experience for patients
		Establish a trusting nurse–patient relationship
	Informational value	Improve patient compliance
		Enhance patient self‐management abilities
		Coordinate various stakeholders
	Social value	Improve public health outcomes
		Promote equitable access to nursing resources
		Disseminate health knowledge to the community
		Advance the nursing profession

Antecedents	System level	Policies and laws
		Social recognition
	Organizational level	Data support
		Human resource management
	Nurse level	Knowledge and experience
		Clinical competence
		Personal attributes

Consequences	Patient‐related	Improve health outcomes
	Nurse‐related	Enhance nurses’ sense of self‐worth
		Increase nurses’ work motivation
	Hospital‐related	Improve hospital service quality
		Enhance hospital reputation
		Achieve value‐based healthcare goals
	Profession‐related	Increase resource investment
		Improve nursing workforce stability
		Elevate the status of the nursing profession
		Advance the nursing profession

##### 3.1.1.1. Technical Value

Nurses apply specialized knowledge and skills to deliver professional procedures and intellectual labor in clinical practice. This value encompasses accurately assessing and identifying nursing issues [23, 24], developing and implementing individualized care plans [23, 24], performing safe and effective technical procedures [25, 26], conducting surveillance and providing early warning [27–29], and delivering comfort care [30].

##### 3.1.1.2. Economic Value

Economic value refers to the contribution of nursing services to cost containment, efficiency improvement, avoidance of preventable resource consumption, and return on health investment. In the reviewed literature, economic value was often reflected through indicators such as shortened recovery time [25, 28, 31], reduced complications [27, 28, 31], lower readmission rates [25, 31, 32], and improved cost‐effectiveness [33–35]. In this study, these indicators were treated as manifestations or consequences of economic value rather than as the attribute itself, so as to distinguish the value dimension from its outcomes.

##### 3.1.1.3. Humanistic Value

Nursing care embodies respect and concern for human life, dignity, and emotions. This involves treating patients with respect [25, 30, 36], providing psychological support [6, 25, 31], creating a positive care experience for patients [14, 28, 37], and establishing a trusting nurse–patient relationship [28, 32, 38].

##### 3.1.1.4. Informational Value

Nursing services function as an information hub for knowledge transfer, coordination, and communication. This is demonstrated through health education that improves patient compliance [25] and enhances self‐management abilities [23, 25, 32], as well as through coordinating various stakeholders [13, 25].

##### 3.1.1.5. Social Value

The societal contributions of nursing services encompass improving public health outcomes [12, 39, 40], promoting equitable access to nursing resources [6, 14, 41], disseminating health knowledge to the community [30], and advancing the nursing profession.

#### 3.1.2. Antecedents of Clinical Nursing Service Value

##### 3.1.2.1. System Level

This includes policy and legal support, social recognition, and respect for the nursing profession. Policies and laws define the role of nursing within the healthcare system and enable the lawful practice of nurses, thereby providing the operational framework necessary for nursing services [6, 39]. Specifically, the method of nursing billing directly impacts how nursing contributions are represented. For instance, bundling nursing fees into bed charges significantly obscures the profession’s unique value [28, 42]. Social recognition and respect for the nursing profession not only influence financial investment in nursing [6] but also affect nurses′ perception of their own professional worth, thereby impacting the overall value of nursing services [14].

##### 3.1.2.2. Organizational Level

This encompasses data support and human resource management. Structured, shareable, and comparable nursing data, coupled with robust nursing information systems, create the necessary conditions for effectively demonstrating and measuring clinical nursing service value [5, 24, 42]. Investment in nursing human capital is a primary driver of value creation, encompassing recruitment, retention, continuing professional education, salaries, incentive measures, and other diversity and inclusion strategies [13, 28]. Assigning reasonable workloads enables nurses to dedicate more time to direct patient contact, thereby enhancing value creation [13, 38, 41].

##### 3.1.2.3. Nurse Level

This involves the nurse’s knowledge and experience, clinical competence, and personal attributes. A nurse’s capacity to create value is linked to their education level, acquired knowledge, and clinical experience [14, 27, 43]. Proficiency in technical skills, clinical reasoning, communication, decision‐making, and leadership is crucial for positive patient outcomes and safety [27, 33, 43]. Personal attributes act as the internal engine for value creation, exemplified by work motivation, self‐perception, emotional engagement, and a sense of ethical responsibility [23, 25, 43].

#### 3.1.3. Consequences of Clinical Nursing Service Value

##### 3.1.3.1. Patient‐Related Consequences

Valuable clinical nursing care improves patient health outcomes [32, 35, 38].

##### 3.1.3.2. Nurse‐Related Consequences

Through delivering valuable care, nurses can perceive their own worth and importance [13, 44], which positively influences their work motivation and professional attitude [45].

##### 3.1.3.3. Hospital‐Related Consequences

Recognizing and enhancing clinical nursing service value helps improve the quality of care and the hospital’s overall service level [11]. Patients satisfied with nursing care become advocates for the hospital, enhancing its reputation and competitiveness [13]. The realization of nursing service value also improves the hospital’s financial management and facilitates the achievement of value‐based healthcare goals [39, 46].

##### 3.1.3.4. Profession‐Related Consequences

When nursing value is demonstrated by individual nurses and hospitals and accumulates into profession‐wide achievements, it transforms perceptions among society and policymakers. This shift helps secure greater stakeholder investment in nursing [13, 28, 34] and elevates nurses’ status within healthcare and society [30, 47]. Conversely, undervaluing nursing services often leaves nurses vulnerable during hospital reforms, causing workforce instability and talent loss [14, 45]. These factors ultimately determine the nursing profession’s sustainable development [14].

#### 3.1.4. Working Definition of Clinical Nursing Service Value

From the literature review, we identified a working definition of clinical nursing service value as follows:

Clinical nursing service value is the sum of positive benefits across technical, economic, humanistic, informational, and social dimensions generated in clinical nursing practice. This value is created by nurses with support from system and organizational factors and through the application of their individual knowledge, competencies, and attributes. Ultimately, it generates impacts on patients, nurses, healthcare institutions, and the nursing profession.

### 3.2. Findings of Fieldwork Phase

From the qualitative data, 12 categories and 37 subcategories emerged, describing the attributes, antecedents, and consequences of clinical nursing services (Tables 4 and 5).

**TABLE 4:** Attributes of clinical nursing service value in the fieldwork phase.

Category	Subcategory
Technical value	Assessment and monitoring value
	Intervention and operational value
	Safety and protection value

Economic value	Cost control value
	Operational efficiency optimization value
	Health investment return value

Humanistic value	Respect and dignity maintenance value
	Emotional support and comfort value
	Communication empowerment and development value

Informational value	Information generation and recording value
	Information transmission and coordination value
	Information interpretation and education value

Social value	Health policy implementation value
	Professional development and promotion value
	Health culture and knowledge dissemination value

**TABLE 5:** Antecedents and consequences of clinical nursing service value in the fieldwork phase.

The phase of the study	Category	Subcategory
Antecedents	System and organization	Policy environment
		Resource guarantee
		Strategic culture
		Process Mechanisms
	Nursing team	Professional competence
		Professional attributes and values
		Leadership and management
	Patient and family	Recognition and respect
		Trust and cooperation
	Technology and information	Technology and information support

Consequences	Patient	Improvement of health outcomes
		Enhancement of medical experience
		Improvement of health literacy
		Improvement of social function
	Nurse and nursing profession	Enhancement of professional fulfillment
		Personal competence development
		Improvement of professional status
		Acquisition of development resources
	Hospital and healthcare system	Improvement of overall hospital service level
		Enhancement of hospital reputation
		Improvement of patient loyalty and recommendation intention
		Enhancement of healthcare system credibility

#### 3.2.1. Attributes of Clinical Nursing Service Value

##### 3.2.1.1. Technical Value

Nursing services deliver professional operations and intellectual labor by applying specialized knowledge and techniques, enabling accurate assessment of health issues, effective implementation of nursing interventions, and ensuring patient safety throughout the entire diagnostic and treatment process. First, nurses conduct continuous assessment and monitoring of patients. When asked about the specific manifestations of the clinical nursing service value, nurses emphasized the critical role of comprehensive assessment and early detection of disease progression.

*“You have to fully assess what the real problem is. Some patients need a stoma, others need long-term management, and some need a better quality of life… Then we provide personalized care. That′s how we truly reflect our value.” (M2)*

*“For example, a patient with carbon dioxide retention might go from being fine to lethargic from one day to the next. We are the first to notice and alert the doctor, since doctors cannot be at the bedside constantly.” (N2)*

Second, the implementation of nursing interventions can address patients′ clinical issues and facilitate disease recovery. The interviewed nurses emphasized the importance of “timeliness in patient care” and “symptom relief.” Patients placed particular importance on procedural comfort, with a focus on “one‐time success” and “painlessness.” Furthermore, nurses ensure patient safety through risk prevention and emergency response measures, such as assessing the need for monitoring devices prior to patient transfer or reviewing medical orders to identify potential errors—these actions constitute critical components of patient safety.

##### 3.2.1.2. Economic Value

Economic value refers to the contribution of nursing services to cost containment, efficiency improvement, avoidance of preventable resource consumption, and return on health investment. This value is reflected not only in reducing unnecessary expenditure but also in improving the efficiency and sustainability of care delivery. Nurses described how timely assessment, risk identification, and early intervention could prevent avoidable deterioration and reduce additional treatment costs. One nurse illustrated this in detail using the example of a digestive fistula:

*“If the fistula spreads to the abdominal cavity, it may require not only double-lumen catheter irrigation but also surgery, resulting in additional costs. Early intervention can definitely reduce costs for patients and the hospital.” (N9)*

Nursing services also generate long‐term economic value by improving patients’ self‐management and preventing disease progression. As one nurse noted, professional guidance on healthy behaviors and tertiary prevention can reduce both household and societal economic burden (N8).

##### 3.2.1.3. Humanistic Value

Nursing care fosters a safe, trusting, and supportive environment by respecting and caring for individuals’ lives, dignity, emotions, and rights, while promoting the positive development of patients’ psychological and social adaptability. For example, when family members request confidentiality regarding the diagnostic results, the nurse emphasizes the need to adjust communication methods while respecting their wishes and ensuring patient safety. In terms of emotional support, the nurses’ empathetic care has helped patients feel more optimistic, leading to improved clinical outcomes. Additionally, nurses establish trust through communication and facilitate the development of social support networks. For example, a head nurse highlighted the effectiveness of establishing a diabetes peer support group:

*“We have established such peer support groups for diabetics… Their compliance and achievement rates are higher. “ (M2)*

##### 3.2.1.4. Informational Value

Nursing services record, transmit, and interpret health data to ensure information continuity and enhance patients’ self‐management. Regarding information recording, nurses objectively and accurately document comprehensive cycle data, including patient admission assessments, disease progression, and discharge satisfaction, facilitating traceability and statistical analysis. In terms of information coordination, nursing coordinates communication among stakeholders to ensure seamless care. A nurse shared an experience in resolving scheduling conflicts for a patient:

*“If a patient has a computed tomography scan at 10:00, but also a dynamic electrocardiogram scheduled for 9:00, that doesn′t work. We immediately coordinate with the electrocardiogram department to resolve it.” (N3)*

Through health education, nursing improves patients’ disease awareness and self‐management. For example, nurses provide one‐on‐one guidance to diabetic patients upon discharge on how to administer insulin injections and determine appropriate dosages.

##### 3.2.1.5. Social Value

Nursing services promote health equity and professional development through policy implementation, research, and health education. Nurses play a foundational role in implementing national health policies. Nurses believe that national policies can be implemented and the social value of nursing realized through specific initiatives such as rehabilitation therapy and weight management. At the level of professional development, nursing practice promotes continuous advancement in both the nursing discipline and the healthcare industry through talent cultivation, model innovation, and the translation of research findings into clinical practice. Nursing services also assume the social responsibility of health culture and knowledge dissemination. A head nurse cited a real‐life case:

*“We previously had a patient… He went to college and was away from parental supervision. Then one day he drank nine bottles of sugary drinks and immediately developed ketoacidosis—it happened that fast. All this is because health education isn′t widespread enough, and we nurses haven′t fully utilized our professional value.” (M2)*

#### 3.2.2. Antecedents of Clinical Nursing Service Value

##### 3.2.2.1. System and Organization Level

This level includes the policy environment, resource guarantee, strategic culture, and processes and systems. The policy environment is the top‐level design determining whether nursing value can be realized. Resource guarantee refers to the human, material, and financial support for nursing services. When asked about the factors influencing value realization, a nurse elaborated on the mechanism of personnel stability:

*“For example, job transfers—our department is relatively stable, but some departments have frequent nurse turnover, which may erode nurses′ sense of belonging and enthusiasm.” (N3)*

The strategic culture of an organization can provide nurses with psychological support and an environment for innovation. Fair and well‐designed procedural mechanisms, such as complaint resolution systems, are crucial for safeguarding nurses′ motivation.

##### 3.2.2.2. Nursing Team Level

This level includes nurses’ professional competence, professional ethics and values, and the leadership of nurse managers. As the direct providers of nursing services, nurses’ knowledge level, clinical competence, and accumulated experience directly influence value creation. A head nurse introduced the requirements for nurses’ clinical capabilities:

*“These capabilities include observation skills, emergency response capabilities, communication skills, and nursing documentation writing skills… On this basis, we then select those with good teaching capabilities, research capabilities, or strong organizational and coordination capabilities.” (M2)*

Nurses’ professional attributes and values are the internal driving force for their value creation. Managers emphasize that nurses must overcome the perception of being “low‐level personnel” through writing and other means, actively demonstrating their personal value. The leadership competence of nurse managers is closely linked to whether nurses can fully realize their value. Experienced head nurses know how to leverage staff strengths to motivate teams and maximize nursing value.

##### 3.2.2.3. Patient and Family Level

This level includes recognition, respect, trust, and cooperation with nurses. The development of the nursing profession is influenced by societal perception. Nurses report that many people hold a dismissive attitude toward them, which hinders the advancement of the nursing profession in certain aspects. A head nurse viewed patient engagement as the source of nursing value:

*“In fact, patients serve as important supervisors who help us improve quality… They can uncover much of our potential value—they are the very source of our value.” (M2)*

##### 3.2.2.4. Technology and Information Level

Nursing services require support from information systems. A head nurse expressed the expectations of clinical nurses regarding the development of information systems:

*“As nurses often point out, the current information system requires too much data entry. What we really want is for the system to help reduce nurses′ workload—for example, by automatically generating routine vital signs and shift reports. This would give nurses more time for direct patient care. We also hope it can document our workload and contributions, making the value of our work visible.” (M1)*

#### 3.2.3. Consequences of Clinical Nursing Service Value

##### 3.2.3.1. Patient‐Related Consequences

The realization of nursing service value leads to improved patient health outcomes, enhanced care experiences, increased health literacy, and improved social functioning. A patient directly highlighted the role of nursing care in rehabilitation:

*“In the end, how well you recover—and how fast—really depends on the nursing care you get.” (P24)*

The patient‐centered explanations provided by nurses enhance patient satisfaction. Furthermore, many patients report adopting healthy lifestyles and actively managing their health after receiving health education. This demonstrates that the value of nursing extends beyond improving clinical outcomes to empowering patients in sustaining long‐term healthy behaviors.

##### 3.2.3.2. Nurse and Nursing Profession‐Related Consequences

Value realization rewards nursing itself, bringing enhanced professional sense of achievement, personal competence development for nurses, and contributing to the improvement of the nursing profession’s status and the acquisition of development resources.

*“The warm conversations with patients, and their heartfelt thanks when we help them—these moments make us feel that this profession is needed by patients.” (N3)*

In the process of creating value, nurses’ personal capabilities are continuously improved. Patients also perceive the professionalism of nursing care through their experience, thereby gradually altering societal stereotypes about nursing and enhancing its professional status. More importantly, this value realization enables the nursing profession to secure greater resource support, including funding, academic guidance, and equipment, further advancing its development.

##### 3.2.3.3. Hospital and Healthcare System‐Related Consequences

The realization of the nursing service value can enhance the overall service quality of hospitals, strengthen their reputation, increase patient loyalty and referral willingness, and improve the credibility of the healthcare system. One patient explicitly described how substandard nursing care affects healthcare‐seeking decisions:

*“Some hospitals have poor nursing service. I stayed at one once. Put yourself in that position—if you′ve been to one like that, you wouldn′t want to go back either.” (P9)*

Conversely, high‐quality nursing care can transform patients′ perceptions of hospitals and enhance trust in nurses, healthcare institutions, and the entire medical system. This trust not only facilitates smoother nursing practices but also constitutes the social foundation for the sustainable development of the healthcare system. As one nurse put it:

*“When nursing is done well, patients trust us more. It′s not just trust in the nurses, but also in the hospital and all the medical services they receive. That trust also makes our work smoother.” (N7)*

### 3.3. Findings of Analytical Phase

The findings from the theoretical and fieldwork phases were generally consistent in their framework for categorizing clinical nursing service value, with the field interviews providing richer and more contextualized details. Integrating findings from both phases indicates that clinical nursing service value is created through the dynamic process of nurse–patient interaction. The attributes of clinical nursing service value are not isolated from each other but are interconnected. For example, the realization of technical value helps build patient trust, thereby enhancing humanistic value, while all dimensions of value ultimately contribute to social value. The consequences of clinical nursing service value may also be associated with its antecedents, potentially forming a feedback cycle of value enhancement. For instance, high clinical nursing service value may be associated with greater resource support for nursing, potentially supporting continued value creation (Figure 2).

**FIGURE 2:**
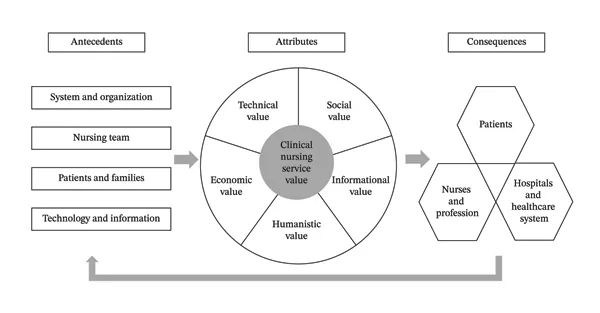
Analytic phase: Integrating the findings from theoretical and fieldwork phase.

Based on this, the concept of clinical nursing service value can be defined as a composite value system dynamically generated through nurse–patient interactions in clinical practice. This system consists of five interconnected dimensions—technical, economic, humanistic, informational, and social. It is associated with factors at the system, organizational, and individual levels and with outcomes concerning patients, nurses, healthcare institutions, and the nursing profession, thereby suggesting a potential value‐creation cycle.

The five dimensions are interrelated but analytically distinct. Technical value refers to nurses’ professional judgment, assessment, intervention, monitoring, and safety protection in direct clinical care. Economic value concerns cost containment, efficiency improvement, avoidance of preventable resource consumption, and return on health investment. Humanistic value captures respect, dignity protection, emotional support, and trust‐building in nurse–patient relationships. Informational value refers to the recording, transmission, coordination, interpretation, and educational use of health information. Social value extends beyond individual care episodes and reflects nursing’s contribution to health equity, public health, professional development, and the dissemination of health knowledge.

## 4. Discussion

This study clarified clinical nursing service value using a hybrid concept analysis model. By integrating literature review and fieldwork interviews, we identified clinical nursing service value as a multidimensional concept comprising technical, economic, humanistic, informational, and social value. The findings extend previous discussions that have mainly emphasized cost‐effectiveness or patient outcomes by making visible the professional judgment, continuous monitoring, coordination, emotional support, health education, and patient engagement embedded in clinical nursing work. A recent concept analysis of value‐based healthcare identified three defining attributes: monetary value of health service, quality of care, and patient‐centered care [26]. In contrast, our study provides a hybrid, multistakeholder extension focused specifically on clinical nursing service value; thus, the two frameworks are complementary.

The main contribution of this study is the clarification of the conceptual boundaries and value‐generating mechanisms of clinical nursing service value. The model distinguishes value attributes from consequences, thereby reducing conceptual ambiguity when outcomes such as recovery, satisfaction, or reduced readmission are treated as the value itself. It also highlights forms of nursing labor that are often hidden in routine care, particularly cognitive labor, informational work, and humanistic care. This provides a conceptual basis for nursing management, performance evaluation, workforce planning, and policy reform.

Our findings showed that clinical nursing service value resides not merely in technical procedures, but is substantially linked to nurses′ ongoing cognitive labor, including judgment, decision‐making, caring, and coordination. Consistent with this finding, one study indicates that nurses undertake decision‐making tasks in clinical practice, such as pain management, triage assessment, intervention selection, and nurse–patient communication. Deviations in these tasks may lead to inadequate information exchange, treatment delays, inappropriate management, or even patient mortality [48]. These cognitive activities permeate the entire nursing process yet are often regarded as invisible work. Although such work consumes considerable nursing time and significantly benefits patient recovery, satisfaction, and safety, it is often overlooked due to measurement challenges [49]. This neglect not only results in underrecognition of nurses’ actual workload and professional value but may also contribute to professional burnout and exert potential negative impacts on the financial performance and quality outcomes of healthcare institutions [50].

During the fieldwork phase of this study, we identified differences in value perceptions between patients and nursing providers. Patients exhibited an experience‐oriented approach to value judgment, placing significant importance on accuracy, timeliness, and comfort within technical value, as well as positive care experiences characterized by humanistic value. Similarly, a previous study indicated that patient experiences in emotional support, health education, and holistic care management are critical factors influencing satisfaction [51]. In contrast, nurses and managers emphasized the demonstration of nursing professionalism and effectiveness across multiple dimensions, including technical care, compassion, and information coordination, while also extending their value perspective to macrolevel concerns such as cost control and professional and social development. Another study also revealed disparities in how patients and nurses perceive nursing behaviors: Patients prioritized knowledge, skill, and safety, whereas nurses valued respect, privacy, and dignity during care delivery. These differences are not contradictory; rather, they reflect the multidimensional nature of nursing service value, suggesting that both patient and nursing perspectives should be considered in strategic nursing decisions [52]. In our study, both patients and nurses acknowledged the importance of communication as a bridge for building trust and conveying health information. However, nurses expressed concerns about insufficient communication, primarily attributing this limitation to staffing constraints that restricted the depth and frequency of interactions. This finding aligns with a previous study that reported communication limitations between nurses and patients due to staffing shortages [53]. Furthermore, our study revealed that some patients had a limited understanding of nursing service value, perceiving nurses primarily as physician assistants and service providers. Such cognitive limitations may become entrenched as societal perceptions, forming cultural antecedents that hinder professional development. The nursing community needs to proactively express and demonstrate its professional value to shape nursing’s professional image within the healthcare system and bridge this cognitive gap.

This study reveals that, in addition to the quality of nursing staff and external support, the proactive engagement of patients and their families is a crucial antecedent in creating nursing service value. Patients’ trust and cooperation enable nurses to better understand their needs, thereby facilitating higher quality care. This finding aligns with the concept of value cocreation, where patients transition from “passive recipients” to “active participants,” collaborating with nurses to jointly create nursing service value [54]. However, the interview findings indicate that most patients currently remain at the stage of passively complying with nursing services, lacking awareness of their role in value creation. Similarly, one study demonstrated that patients’ recognition of the importance of being “treated as individuals” or “participating in care planning” is significantly lower than that of nursing staff, ranking these aspects last in their prioritization of nursing behaviors [52]. Active patient participation in creating clinical nursing service value can improve patients’ quality of life and reduce hospital stay duration [55]. In turn, nurses can achieve higher job satisfaction through such positive interactions, leading to better patient health outcomes, more efficient service resource allocation, and an enhanced care environment [56]. Therefore, enhancing patients′ awareness and capability for active participation in value cocreation may represent a key pathway to maximizing nursing service value.

This study further suggests that clinical nursing service value may operate as a dynamic system with feedback loops, characterized by possible interactions between its antecedents and consequences. The successful realization of value may reciprocally reinforce its antecedents, potentially establishing a self‐reinforcing cyclical mechanism. These proposed relationships are derived from the integrated findings of the literature review and fieldwork and require subsequent empirical testing to establish their validity and generalizability. For instance, when nursing services fully demonstrate their social and economic value, they can secure greater policy and resource support for the profession, thereby optimizing the supportive environment at system and organizational levels. This dynamic perspective aligns with theoretical viewpoints from complexity science that emphasize how healthcare systems function as complex adaptive systems. Their operational mechanisms are not based on simple linear causality but emerge from continuous interactions among multiple agents within the system, generating emergent behaviors that in turn reshape the system’s own structure and rules, forming an ongoing evolutionary process [57]. This dynamic perspective indicates that enhancing nursing service value is not a one‐time investment but rather a strategic undertaking requiring continuous optimization of the supportive environment while demonstrating value to secure better conditions. Establishing such a virtuous cycle mechanism holds crucial significance for achieving sustainable development of nursing service value.

## 5. Conclusion

This study employed a hybrid concept analysis to extend and refine the conceptualization of clinical nursing service value. This model not only provides theoretical clarification of nursing’s core contributions but also offers an integrative framework to guide management and policy decisions, encouraging a more comprehensive and dynamic perspective when evaluating nursing’s professional impact. It positions nursing services as essential health assets worthy of systematic investment. We anticipate that our findings will facilitate the quantitative assessment of nursing service value and exploration of value realization pathways, thereby optimizing nursing care, improving healthcare resource allocation, and ultimately supporting the establishment of a value‐driven healthcare system. Further empirical validation is needed before the model is applied in nursing management practice.

## 6. Implications for Nursing Management

The five‐dimensional model provides a conceptual framework for recognizing the multidimensional contribution of nursing. Once empirically validated, it may inform performance evaluation, workforce planning, resource allocation, and nursing data systems. At present, the model should not be used directly for performance appraisal, reimbursement, or compensation decisions. Future research should develop and validate measurement tools based on this framework.

## 7. Limitations

Several limitations should be acknowledged. First, although the theoretical phase included international literature, the fieldwork data were collected only in China; therefore, the transferability of the model to other healthcare systems requires further validation. Second, participants were recruited from two tertiary Grade A hospitals in East China. Although maximum variation was pursued across departments, roles, professional titles, and years of experience, variation across hospital levels and institutional types was not achieved. Future studies should include primary hospitals, community health centers, long‐term care institutions, and home‐based nursing settings. Third, the nurse sample included a relatively high proportion of experienced nurses, which may have limited the representation of novice nurses’ perspectives. Fourth, saturation was assessed across the complete sample rather than separately within participant subgroups, and only three nurse managers were included. Therefore, the managerial perspective requires further exploration. Finally, the five‐dimensional model remains a conceptual framework requiring empirical validation before its use in performance appraisal, reimbursement, or compensation decisions.

## Funding

This work was supported by the Open Research Project on Nursing Discipline, Nanjing University of Chinese Medicine Jiangsu Higher Education Institutions Advantage Discipline Phase IV Project (Grant Nos. YSHL202503, YSHL202528); and Postgraduate Research & Practice Innovation Program of Jiangsu Province (Grant No. SJCX25_1112).

## Conflicts of Interest

The authors declare no conflicts of interest.

## Supporting Information

Additional supporting information can be found online in the Supporting Information section.

## Supporting information


**Supporting Information** Supporting File S1: PRISMA 2020 and COREQ Checklists. Supporting File S2: Literature search strategies. Supporting File S3: Data extraction form for literature review. Supporting File S4: An example of data analysis for Clinical nursing service value.

## Data Availability

The data that support the findings of this study are available from the corresponding author upon reasonable request.
