# Supplementary material for: Clinical Nursing Service Value: A Hybrid Concept Analysis
Source: J Nurs Manag. 2026 Oct 23;2026:7028770. doi: 10.1155/jonm/7028770 (PMC13691524; doi:10.1155/jonm/7028770)
Supplement: Supplementary file 1 [file JONM-2026-7028770-s001.zip › Revised-Supplementary File S2 Literature search strategies.docx]

**Clinical Nursing Service Value: A Hybrid Concept Analysis**

**Literature search strategies**

Table 1. PubMed	Search date: September 14, 2026

| # | Searches | Results |
| --- | --- | --- |
| #1 | "Nursing"[Mesh] | 275,061 |
| #2 | "Nurses"[Mesh] | 106,915 |
| #3 | nurs*[Title/Abstract] OR care[Title/Abstract] | 2,692,674 |
| #4 | #1 OR #3 OR #2 | 2,815,479 |
| #5 | service value[Title/Abstract] | 500 |
| #6 | #4 AND #5 | 42 |
| #7 | nurs*[Title] AND value[Title] | 1299 |
| #8 | #6 OR #7 | 1338 |

Table 2. Web Of Science	Search date: September 14, 2026

| # | Searches | Results |
| --- | --- | --- |
| #1 | (TI=(nurs* OR care)) OR AB=(nurs* OR care) | 5,215,857 |
| #2 | (TI=("service value")) OR AB=("service value") | 7,714 |
| #3 | #1 AND #2 | 164 |
| #4 | (TI=(nurs*)) AND TI=(value) | 4,575 |
| #5 | #3 OR #4 | 4,732 |

Table 3. MEDLINE Complete	Search date: September 14, 2026

| # | Searches | Results |
| --- | --- | --- |
| S1 | TI ( nurs* OR care ) OR AB ( nurs* OR care ) | 2,653,738 |
| S2 | TI "service value" OR AB "service value" | 476 |
| S3 | S1 AND S2 | 38 |
| S4 | TI nurs* AND TI value | 2068 |
| S5 | S3 OR S4 | 2103 |

Table 4. CINAHL Complete	Search date: September 14, 2026

| # | Searches | Results |
| --- | --- | --- |
| S1 | TI ( nurs* OR care ) OR AB ( nurs* OR care ) | 1,545,043 |
| S2 | TI "service value" OR AB "service value" | 42 |
| S3 | S1 AND S2 | 21 |
| S4 | TI nurs* AND TI value | 2603 |
| S5 | S3 OR S4 | 2621 |

Table 5. Embase	Search date: September 14, 2026

| # | Searches | Results |
| --- | --- | --- |
| #1 | nurse:ti,ab,kw OR nursing:ti,ab,kw OR care:ti,ab,kw | 3,713,506 |
| #2 | 'service value':ti,ab,kw | 509 |
| #3 | #1 AND #2 | 60 |
| #4 | nursing:ti OR nurse:ti | 268,508 |
| #5 | value:ti | 233,498 |
| #6 | #4 AND #5 | 1162 |
| #7 | #3 OR #6 | 1219 |

Table 6. Emerald Insight	Search date: September 14, 2026

| # | Searches | Results |
| --- | --- | --- |
| 1 | ((title:(nurs* OR care) OR abstract:(nurs* OR care)) AND "service value") OR (title:nurs* AND title:value) | 2498 |

Table 7. China National Knowledge Infrastructure	Search date: September 14, 2026

| # | Searches | Results |
| --- | --- | --- |
| 1 | TKA=('nursing'+'nurse'+'nursing service') AND TKA=('service value'+'professional value') | 782 |

Table 8. Wanfang Data Knowledge Service Platform	Search date: September 14, 2026

| # | Searches | Results |
| --- | --- | --- |
| 1 | (Title or keywords:("nursing" or "nurse" or "nursing service") or Abstract:("nursing" or "nurse" or "nursing service")) and (Title or keywords:("service value" or "professional value") or Abstract:("service value" or "professional value")) | 901 |

Table 9. Chinese Science and Technology Journals Database Search date: September 14, 2026

| # | Searches | Results |
| --- | --- | --- |
| 1 | M=(“nursing” OR “nurse” OR “nursing service”) AND M=(“service value” OR “professional value”) | 522 |

Table 10. China Biology Medicine disc	Search date: September 14, 2026

| # | Searches | Results |
| --- | --- | --- |
| 1 | ("nursing"[Common fields: Smart] OR "nurse"[Common fields: Smart] OR "nursing service"[Common fields: Smart]) AND ("service value"[Common fields: Smart] OR "professional value"[Common fields: Smart]) | 556 |
