# Supplementary material for: Clinical Nursing Service Value: A Hybrid Concept Analysis
Source: J Nurs Manag. 2026 Oct 23;2026:7028770. doi: 10.1155/jonm/7028770 (PMC13691524; doi:10.1155/jonm/7028770)
Supplement: Supplementary file 1 [file JONM-2026-7028770-s001.zip › Supplementary Flie S4 An example of data analysis in Clinical nursing service value..docx]

**Clinical Nursing Service Value: A Hybrid Concept Analysis**

**SUPPLEMENTARY TABLE: An example of data analysis in Clinical nursing service value.**

| Meaning unit | Codes | Subcategories | Categories |
| --- | --- | --- | --- |
| Due to the more frequent contact between nursing staff and patients compared to medical professionals, nursing can be regarded as a sentinel system, as it enables early detection of potential issues for timely reporting and warning. | Risk monitoring and Early warning | Assessment and monitoring value | Technical value |
